# 3-[2-(6-Bromo-2-phenyl-3*H*-imidazo[4,5-*b*]pyridin-3-yl)eth­yl]-1,3-oxazolidin-2-one

**DOI:** 10.1107/S1600536811012669

**Published:** 2011-04-13

**Authors:** Younes Ouzidan, Jerry P. Jasinski, Raymond J. Butcher, James A. Golen, El Mokhtar Essassi, Lahcen El Ammari

**Affiliations:** a Laboratoire de Chimie Organique Appliquée, Université Sidi Mohamed Ben Abdallah, Faculté des Sciences et Techniques, Route d’Immouzzer, BP 2202 Fès, Morocco; b Department of Chemistry, Keene State College, 229 Main Street, Keene, NH 03435-2001, USA; c Department of Chemistry, Howard University, 525 College Street NW, Washington, DC 20059, USA; d Laboratoire de Chimie Organique Hétérocyclique, URAC 21, Avenue Ibn Battouta, Rabat, Morocco; e Laboratoire de Chimie du Solide Appliquée, Faculté des Sciences, Université Mohammed V-Agdal, Avenue Ibn Battouta, BP 1014, Rabat, Morocco

## Abstract

In the title mol­ecule, C_17_H_15_BrN_4_O_2_, the fused-ring system is essentially planar, the largest deviation from the mean plane being 0.015 (2) Å, and forms dihedral angles of 37.8 (2) and 35.5 (2)° with the phenyl and oxazolidine rings, respectively. The conformation adopted by the mol­ecule is stabilized by an intra­molecular π⋯π inter­action [centroid–centroid distance = 3.855(2) Å] between oxazolidine and phenyl rings. The crystal packing features inter­molecular C—H⋯N and C—H⋯O inter­actions.

## Related literature

For background to the medicinal chemistry of oxazolidin-2-ones and their application in asymmetric synthesis, see: Diekema & Jones (2000 ▶); Mukhtar & Wright (2004 ▶); Evans *et al.* (1993 ▶); Matsunaga *et al.* (2005 ▶). For similar compounds with an imidazo[4,5-*b*]pyridine group, see: Ouzidan *et al.* (2010*a* ▶,*b* ▶).
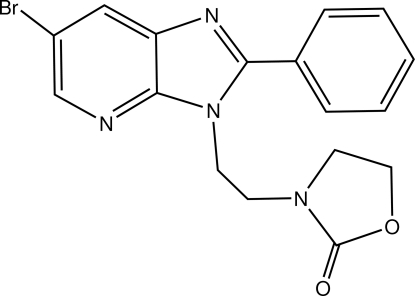


## Experimental

### 

#### Crystal data

C_17_H_15_BrN_4_O_2_
*M* *_r_* = 387.24
Monoclinic, 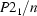

*a* = 11.3553 (6) Å
*b* = 11.5915 (5) Å
*c* = 12.2542 (8) Å
β = 98.685 (6)°
*V* = 1594.46 (15) Å^3^
*Z* = 4
Mo *K*α radiation
μ = 2.60 mm^−1^
*T* = 170 K
0.22 × 0.20 × 0.18 mm

#### Data collection

Oxford Diffraction XcaliburE Gemini diffractometer
Absorption correction: multi-scan (*CrysAlis PRO*; Oxford Diffraction, 2009 ▶) *T*_min_ = 0.599, *T*_max_ = 0.652
7850 measured reflections
3791 independent reflections
2839 reflections with *I* > 2σ(*I*)
*R*_int_ = 0.028

#### Refinement

*R*[*F* ^2^ > 2σ(*F* ^2^)] = 0.037
*wR*(*F* ^2^) = 0.091
*S* = 1.03
3791 reflections
218 parameters
H-atom parameters constrained
Δρ_max_ = 0.50 e Å^−3^
Δρ_min_ = −0.35 e Å^−3^

### 

Data collection: *CrysAlis PRO* (Oxford Diffraction, 2009 ▶); cell refinement: *CrysAlis PRO*; data reduction: *CrysAlis PRO*; program(s) used to solve structure: *SHELXS97* (Sheldrick, 2008 ▶); program(s) used to refine structure: *SHELXL97* (Sheldrick, 2008 ▶); molecular graphics: *SHELXTL* (Sheldrick, 2008 ▶); software used to prepare material for publication: *SHELXTL*.

## Supplementary Material

Crystal structure: contains datablocks I, global. DOI: 10.1107/S1600536811012669/gk2362sup1.cif

Structure factors: contains datablocks I. DOI: 10.1107/S1600536811012669/gk2362Isup2.hkl

Additional supplementary materials:  crystallographic information; 3D view; checkCIF report

## Figures and Tables

**Table 1:** Hydrogen-bond geometry (Å, °)

*D*—H⋯*A*	*D*—H	H⋯*A*	*D*⋯*A*	*D*—H⋯*A*
C4—H4*A*⋯N2^i^	0.95	2.61	3.544 (3)	168
C10—H10*A*⋯N2^ii^	0.99	2.55	3.261 (3)	128
C15—H15*A*⋯O1^iii^	0.95	2.53	3.423 (3)	156

Symmetry codes: (i) 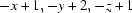; (ii) 
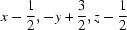
; (iii) 
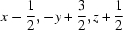
.

**Fig. 1.**
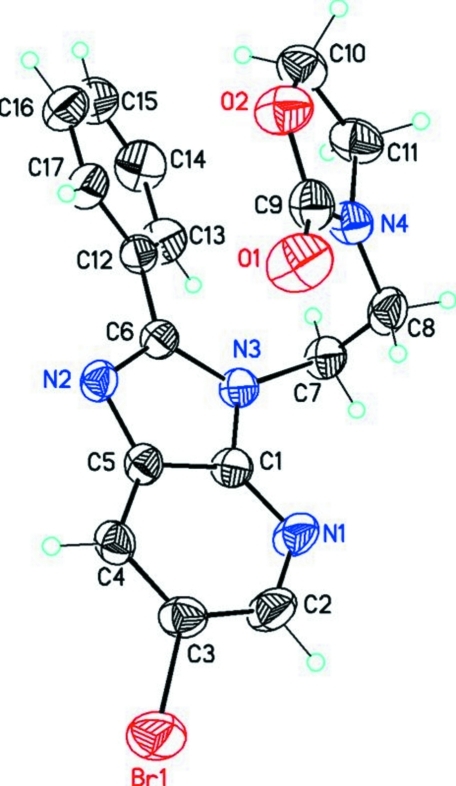
: Molecular structure of the title compound with displacement ellipsoids drawn at the 50% probability level. H atoms are represented as small circles.

**Fig. 2.**
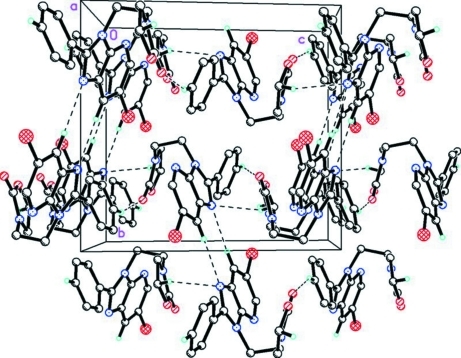
: Partial packing view showing the C—H···O and C—H···N interactions (dashed lines).

## supplementary crystallographic information

### Comment

Oxazolidin-2-ones are a very important class of heterocyclic compounds and their derivatives have attracted attention in various areas of drug development for antibacterial activity (Diekema & Jones, 2000; Mukhtar & Wright, 2004). Some oxazolidin-2-ones have been used as chiral auxiliaries in a wide range of asymmetric reactions (Evans *et al.*,1993; Matsunaga *et al.*, 2005). As a continuation of our research works devoted to the development of substituted imidazo[4,5-*b*]pyridine derivatives (Ouzidan *et al.*, 2010*a*,*b*), we report in this paper the synthesis of a new 2,6-disubstituted imidazo[4,5-*b*]pyridine possessing the oxazolidin-2-one ring (Scheme 1) by the action of bis(2-chloroethyl)amine hydrochloride on 6-bromo-2-phenyl-3*H*-imidazo [4,5-*b*]pyridine in boiling DMF.

The title molecule is shown in Fig. 1. The two cycles forming the imidazo[4,5-*b*]pyridine are almost planar with a maximum deviation of 0.015 (1) Å for N3 atom and form dihedral angles of 37.8 (2)° and 35.5 (2)° with the phenyl and the oxazolidine rings respectively. The oxazolidinone group is linked to the phenyl group by a weak intramolecular C-H···π interaction. The crystal structure is stabilized by two intermolecular C–H···N and C–H···O interactions as shown in Fig. 2 and Table 2.

### Experimental

To 6-bromo-2-phenyl-3*H*-imidazo[4,5-*b*]pyridine (0.3 g, 1.09 mmol), potassium carbonate (0.33 g, 2.4 mmol) and tetra-n-butylammonium bromide (0.05 g, 0.1 mmol) in DMF (15 ml) was added bis(2-chloroethyl)amine hydrochloride (0.23 g, 1.31 mmol). The mixture was heated for 48 h. After the completion of the reaction (as monitored by TLC), the inorganic salt was filtered and the solvent was removed under reduced pressure. The residue was purified by column chromatography on silica gel by using (ethanol/ethyl acetate: 1/4) as eluent. The product was recrystallized from ethanol to furnish colourless crystals (m.p. 454 K).

### Refinement

All H atoms were located in a difference map. They were refined in a riding model approximation with C—H = 0.95 -0.99 Å and *U*_iso_(H) = 1.2 *U*_eq_(C).

### Crystal data

**Table:**

C_17_H_15_BrN_4_O_2_	*F*(000) = 784
*M**_r_* = 387.24	*D*_x_ = 1.613 Mg m^−^^3^
Monoclinic, *P*2_1_/*n*	Mo *K*α radiation, λ = 0.71073 Å
Hall symbol: -P 2yn	Cell parameters from 3008 reflections
*a* = 11.3553 (6) Å	θ = 3.5–32.3°
*b* = 11.5915 (5) Å	µ = 2.60 mm^−^^1^
*c* = 12.2542 (8) Å	*T* = 170 K
β = 98.685 (6)°	Block, colourless
*V* = 1594.46 (15) Å^3^	0.22 × 0.20 × 0.18 mm
*Z* = 4	

### Data collection

**Table:**

Oxford Diffraction XcaliburE Gemini diffractometer	3791 independent reflections
Radiation source: Enhance (Mo) X-ray Source	2839 reflections with *I* > 2σ(*I*)
graphite	*R*_int_ = 0.028
Detector resolution: 16.1500 pixels mm^-1^	θ_max_ = 27.9°, θ_min_ = 3.5°
ω scans	*h* = −9→14
Absorption correction: multi-scan (*CrysAlis PRO*; Oxford Diffraction, 2009)	*k* = −15→6
*T*_min_ = 0.599, *T*_max_ = 0.652	*l* = −15→16
7850 measured reflections	

### Refinement

**Table:**

Refinement on *F*^2^	Secondary atom site location: difference Fourier map
Least-squares matrix: full	Hydrogen site location: inferred from neighbouring sites
*R*[*F*^2^ > 2σ(*F*^2^)] = 0.037	H-atom parameters constrained
*wR*(*F*^2^) = 0.091	*w* = 1/[σ^2^(*F*_o_^2^) + (0.0373*P*)^2^ + 0.4211*P*] where *P* = (*F*_o_^2^ + 2*F*_c_^2^)/3
*S* = 1.03	(Δ/σ)_max_ = 0.001
3791 reflections	Δρ_max_ = 0.50 e Å^−^^3^
218 parameters	Δρ_min_ = −0.35 e Å^−^^3^
0 restraints	Extinction correction: *SHELXL97* (Sheldrick, 2008), Fc^*^=kFc[1+0.001xFc^2^λ^3^/sin(2θ)]^-1/4^
Primary atom site location: structure-invariant direct methods	Extinction coefficient: 0.0047 (7)

### Special details

**Table:**

Geometry. All e.s.d.'s (except the e.s.d. in the dihedral angle between two l.s. planes) are estimated using the full covariance matrix. The cell e.s.d.'s are taken into account individually in the estimation of e.s.d.'s in distances, angles and torsion angles; correlations between e.s.d.'s in cell parameters are only used when they are defined by crystal symmetry. An approximate (isotropic) treatment of cell e.s.d.'s is used for estimating e.s.d.'s involving l.s. planes.
Refinement. Refinement of *F*^2^ against ALL reflections. The weighted *R*-factor *wR* and goodness of fit *S* are based on *F*^2^, conventional *R*-factors *R* are based on *F*, with *F* set to zero for negative *F*^2^. The threshold expression of *F*^2^ > σ(*F*^2^) is used only for calculating *R*-factors(gt) *etc*. and is not relevant to the choice of reflections for refinement. *R*-factors based on *F*^2^ are statistically about twice as large as those based on *F*, and *R*- factors based on ALL data will be even larger.

### Fractional atomic coordinates and isotropic or equivalent isotropic displacement parameters (Å^2^)

**Table:**

	*x*	*y*	*z*	*U*_iso_*/*U*_eq_	
Br1	0.83535 (2)	0.94591 (2)	0.34797 (3)	0.05968 (13)	
O1	0.28122 (17)	0.80153 (15)	0.18265 (18)	0.0643 (6)	
O2	0.09323 (15)	0.76454 (14)	0.20646 (16)	0.0521 (5)	
N1	0.61867 (17)	0.66117 (16)	0.33964 (18)	0.0444 (5)	
N2	0.42350 (16)	0.82558 (14)	0.48010 (15)	0.0331 (4)	
N3	0.43353 (16)	0.64555 (14)	0.41210 (15)	0.0341 (4)	
N4	0.22330 (16)	0.62233 (15)	0.23253 (16)	0.0380 (4)	
C1	0.53419 (19)	0.70349 (17)	0.39157 (18)	0.0336 (5)	
C2	0.7057 (2)	0.7361 (2)	0.3310 (2)	0.0476 (6)	
H2A	0.7694	0.7119	0.2943	0.057*	
C3	0.7086 (2)	0.84809 (19)	0.3727 (2)	0.0386 (5)	
C4	0.61915 (19)	0.89053 (18)	0.42593 (18)	0.0347 (5)	
H4A	0.6204	0.9668	0.4544	0.042*	
C5	0.52729 (19)	0.81425 (17)	0.43494 (18)	0.0313 (5)	
C6	0.37023 (19)	0.72409 (17)	0.46453 (17)	0.0314 (5)	
C7	0.3943 (2)	0.53819 (17)	0.3543 (2)	0.0397 (5)	
H7A	0.3378	0.4977	0.3952	0.048*	
H7B	0.4640	0.4872	0.3526	0.048*	
C8	0.3343 (2)	0.56137 (19)	0.2369 (2)	0.0427 (6)	
H8A	0.3888	0.6072	0.1981	0.051*	
H8B	0.3195	0.4870	0.1975	0.051*	
C9	0.2077 (2)	0.7339 (2)	0.2053 (2)	0.0422 (6)	
C10	0.0290 (2)	0.6685 (2)	0.2428 (2)	0.0473 (6)	
H10A	−0.0444	0.6533	0.1900	0.057*	
H10B	0.0066	0.6841	0.3164	0.057*	
C11	0.1133 (2)	0.5666 (2)	0.2479 (2)	0.0494 (6)	
H11A	0.1207	0.5270	0.3201	0.059*	
H11B	0.0873	0.5104	0.1882	0.059*	
C12	0.25728 (19)	0.69848 (18)	0.50368 (17)	0.0331 (5)	
C13	0.2346 (2)	0.5929 (2)	0.5501 (2)	0.0491 (6)	
H13A	0.2927	0.5333	0.5559	0.059*	
C14	0.1272 (3)	0.5745 (2)	0.5880 (2)	0.0582 (7)	
H14A	0.1116	0.5019	0.6188	0.070*	
C15	0.0434 (2)	0.6598 (3)	0.5815 (2)	0.0572 (7)	
H15A	−0.0304	0.6462	0.6069	0.069*	
C16	0.0661 (2)	0.7651 (2)	0.5383 (2)	0.0511 (6)	
H16A	0.0085	0.8249	0.5350	0.061*	
C17	0.1725 (2)	0.7849 (2)	0.49934 (19)	0.0407 (5)	
H17A	0.1875	0.8581	0.4695	0.049*	

### Atomic displacement parameters (Å^2^)

**Table:**

	*U*^11^	*U*^22^	*U*^33^	*U*^12^	*U*^13^	*U*^23^
Br1	0.04820 (18)	0.05086 (18)	0.0845 (3)	−0.01581 (12)	0.02468 (15)	−0.00566 (14)
O1	0.0624 (12)	0.0360 (9)	0.0993 (16)	−0.0107 (9)	0.0285 (11)	0.0095 (10)
O2	0.0458 (10)	0.0376 (9)	0.0730 (13)	0.0032 (8)	0.0094 (9)	0.0075 (8)
N1	0.0396 (11)	0.0275 (9)	0.0690 (14)	0.0033 (8)	0.0182 (10)	−0.0068 (9)
N2	0.0361 (10)	0.0268 (9)	0.0377 (10)	−0.0006 (8)	0.0093 (8)	−0.0039 (7)
N3	0.0339 (10)	0.0215 (8)	0.0468 (11)	−0.0001 (7)	0.0063 (8)	−0.0022 (8)
N4	0.0378 (11)	0.0287 (9)	0.0472 (12)	−0.0055 (8)	0.0061 (9)	0.0012 (8)
C1	0.0321 (11)	0.0243 (10)	0.0440 (13)	0.0016 (9)	0.0038 (9)	0.0011 (9)
C2	0.0388 (13)	0.0367 (12)	0.0708 (18)	0.0047 (11)	0.0200 (12)	−0.0055 (12)
C3	0.0328 (12)	0.0334 (11)	0.0498 (14)	−0.0021 (9)	0.0071 (10)	0.0032 (10)
C4	0.0375 (12)	0.0246 (10)	0.0412 (13)	−0.0019 (9)	0.0033 (9)	−0.0021 (9)
C5	0.0345 (11)	0.0241 (10)	0.0349 (12)	0.0018 (8)	0.0039 (9)	0.0000 (8)
C6	0.0333 (11)	0.0263 (10)	0.0342 (12)	0.0024 (9)	0.0034 (9)	0.0015 (9)
C7	0.0419 (13)	0.0184 (10)	0.0593 (16)	0.0013 (9)	0.0094 (11)	−0.0036 (9)
C8	0.0491 (14)	0.0297 (11)	0.0517 (15)	−0.0020 (10)	0.0154 (11)	−0.0099 (10)
C9	0.0505 (15)	0.0314 (12)	0.0450 (14)	−0.0046 (11)	0.0084 (11)	−0.0009 (10)
C10	0.0428 (14)	0.0531 (15)	0.0459 (15)	−0.0086 (12)	0.0063 (11)	0.0027 (12)
C11	0.0421 (14)	0.0404 (14)	0.0644 (18)	−0.0132 (11)	0.0037 (12)	0.0041 (12)
C12	0.0341 (11)	0.0336 (11)	0.0319 (12)	−0.0022 (9)	0.0057 (9)	−0.0017 (9)
C13	0.0509 (15)	0.0385 (13)	0.0595 (17)	0.0001 (11)	0.0140 (12)	0.0078 (12)
C14	0.0626 (18)	0.0510 (16)	0.0657 (19)	−0.0159 (14)	0.0248 (15)	0.0080 (13)
C15	0.0475 (16)	0.0679 (18)	0.0607 (18)	−0.0146 (14)	0.0228 (13)	−0.0063 (14)
C16	0.0446 (14)	0.0599 (16)	0.0514 (16)	0.0073 (13)	0.0152 (12)	−0.0050 (13)
C17	0.0453 (13)	0.0374 (12)	0.0418 (13)	0.0017 (10)	0.0139 (10)	−0.0003 (10)

### Geometric parameters (Å, °)

**Table:**

Br1—C3	1.892 (2)	C7—C8	1.520 (4)
O1—C9	1.208 (3)	C7—H7A	0.9900
O2—C9	1.349 (3)	C7—H7B	0.9900
O2—C10	1.438 (3)	C8—H8A	0.9900
N1—C1	1.323 (3)	C8—H8B	0.9900
N1—C2	1.332 (3)	C10—C11	1.516 (4)
N2—C6	1.323 (3)	C10—H10A	0.9900
N2—C5	1.382 (3)	C10—H10B	0.9900
N3—C6	1.378 (3)	C11—H11A	0.9900
N3—C1	1.381 (3)	C11—H11B	0.9900
N3—C7	1.467 (3)	C12—C17	1.385 (3)
N4—C9	1.340 (3)	C12—C13	1.390 (3)
N4—C8	1.439 (3)	C13—C14	1.385 (4)
N4—C11	1.443 (3)	C13—H13A	0.9500
C1—C5	1.396 (3)	C14—C15	1.366 (4)
C2—C3	1.393 (3)	C14—H14A	0.9500
C2—H2A	0.9500	C15—C16	1.370 (4)
C3—C4	1.378 (3)	C15—H15A	0.9500
C4—C5	1.385 (3)	C16—C17	1.383 (3)
C4—H4A	0.9500	C16—H16A	0.9500
C6—C12	1.466 (3)	C17—H17A	0.9500
			
C9—O2—C10	109.50 (18)	N4—C8—H8B	109.0
C1—N1—C2	113.39 (19)	C7—C8—H8B	109.0
C6—N2—C5	104.87 (17)	H8A—C8—H8B	107.8
C6—N3—C1	105.55 (16)	O1—C9—N4	127.8 (2)
C6—N3—C7	130.08 (18)	O1—C9—O2	122.1 (2)
C1—N3—C7	121.58 (18)	N4—C9—O2	110.1 (2)
C9—N4—C8	124.4 (2)	O2—C10—C11	105.60 (19)
C9—N4—C11	112.3 (2)	O2—C10—H10A	110.6
C8—N4—C11	123.08 (18)	C11—C10—H10A	110.6
N1—C1—N3	125.98 (19)	O2—C10—H10B	110.6
N1—C1—C5	127.6 (2)	C11—C10—H10B	110.6
N3—C1—C5	106.39 (18)	H10A—C10—H10B	108.8
N1—C2—C3	123.7 (2)	N4—C11—C10	101.52 (18)
N1—C2—H2A	118.1	N4—C11—H11A	111.5
C3—C2—H2A	118.1	C10—C11—H11A	111.5
C4—C3—C2	121.8 (2)	N4—C11—H11B	111.5
C4—C3—Br1	119.65 (16)	C10—C11—H11B	111.5
C2—C3—Br1	118.43 (17)	H11A—C11—H11B	109.3
C3—C4—C5	115.34 (19)	C17—C12—C13	118.8 (2)
C3—C4—H4A	122.3	C17—C12—C6	118.60 (19)
C5—C4—H4A	122.3	C13—C12—C6	122.5 (2)
N2—C5—C4	132.04 (18)	C14—C13—C12	119.9 (2)
N2—C5—C1	109.89 (18)	C14—C13—H13A	120.0
C4—C5—C1	118.07 (19)	C12—C13—H13A	120.0
N2—C6—N3	113.29 (18)	C15—C14—C13	120.7 (2)
N2—C6—C12	122.43 (19)	C15—C14—H14A	119.7
N3—C6—C12	124.24 (18)	C13—C14—H14A	119.7
N3—C7—C8	111.52 (17)	C14—C15—C16	119.8 (2)
N3—C7—H7A	109.3	C14—C15—H15A	120.1
C8—C7—H7A	109.3	C16—C15—H15A	120.1
N3—C7—H7B	109.3	C15—C16—C17	120.4 (2)
C8—C7—H7B	109.3	C15—C16—H16A	119.8
H7A—C7—H7B	108.0	C17—C16—H16A	119.8
N4—C8—C7	112.74 (19)	C16—C17—C12	120.3 (2)
N4—C8—H8A	109.0	C16—C17—H17A	119.8
C7—C8—H8A	109.0	C12—C17—H17A	119.8
			
C2—N1—C1—N3	179.8 (2)	C1—N3—C7—C8	−76.4 (3)
C2—N1—C1—C5	0.7 (4)	C9—N4—C8—C7	106.1 (3)
C6—N3—C1—N1	−178.2 (2)	C11—N4—C8—C7	−79.5 (3)
C7—N3—C1—N1	−15.3 (3)	N3—C7—C8—N4	−66.7 (2)
C6—N3—C1—C5	1.1 (2)	C8—N4—C9—O1	−1.6 (4)
C7—N3—C1—C5	163.93 (19)	C11—N4—C9—O1	−176.5 (3)
C1—N1—C2—C3	0.3 (4)	C8—N4—C9—O2	178.6 (2)
N1—C2—C3—C4	−0.7 (4)	C11—N4—C9—O2	3.7 (3)
N1—C2—C3—Br1	−177.4 (2)	C10—O2—C9—O1	−176.5 (2)
C2—C3—C4—C5	0.2 (3)	C10—O2—C9—N4	3.3 (3)
Br1—C3—C4—C5	176.72 (16)	C9—O2—C10—C11	−8.5 (3)
C6—N2—C5—C4	179.9 (2)	C9—N4—C11—C10	−8.5 (3)
C6—N2—C5—C1	0.3 (2)	C8—N4—C11—C10	176.5 (2)
C3—C4—C5—N2	−178.8 (2)	O2—C10—C11—N4	9.8 (3)
C3—C4—C5—C1	0.8 (3)	N2—C6—C12—C17	36.7 (3)
N1—C1—C5—N2	178.3 (2)	N3—C6—C12—C17	−145.7 (2)
N3—C1—C5—N2	−0.9 (2)	N2—C6—C12—C13	−139.9 (2)
N1—C1—C5—C4	−1.3 (4)	N3—C6—C12—C13	37.7 (3)
N3—C1—C5—C4	179.46 (19)	C17—C12—C13—C14	1.9 (4)
C5—N2—C6—N3	0.4 (2)	C6—C12—C13—C14	178.5 (2)
C5—N2—C6—C12	178.28 (19)	C12—C13—C14—C15	−0.8 (4)
C1—N3—C6—N2	−1.0 (2)	C13—C14—C15—C16	−0.7 (5)
C7—N3—C6—N2	−161.8 (2)	C14—C15—C16—C17	1.1 (4)
C1—N3—C6—C12	−178.78 (19)	C15—C16—C17—C12	0.0 (4)
C7—N3—C6—C12	20.4 (3)	C13—C12—C17—C16	−1.5 (4)
C6—N3—C7—C8	81.7 (3)	C6—C12—C17—C16	−178.2 (2)

### Hydrogen-bond geometry (Å, °)

**Table:**

*D*—H···*A*	*D*—H	H···*A*	*D*···*A*	*D*—H···*A*
C4—H4A···N2^i^	0.95	2.61	3.544 (3)	168
C10—H10A···N2^ii^	0.99	2.55	3.261 (3)	128
C15—H15A···O1^iii^	0.95	2.53	3.423 (3)	156

Symmetry codes: (i) −*x*+1, −*y*+2, −*z*+1; (ii) *x*−1/2, −*y*+3/2, *z*−1/2; (iii) *x*−1/2, −*y*+3/2, *z*+1/2.
